# Inventing hyperaccumulator plants: improving practice in phytoextraction research and terminology

**DOI:** 10.1080/15226514.2024.2322631

**Published:** 2024-03-04

**Authors:** Antony van der Ent, Elizabeth L. Rylott

**Affiliations:** a Laboratory of Genetics, Wageningen University and Research, Wageningen, The Netherlands; b Centre for Mined Land Rehabilitation, Sustainable Minerals Institute, The University of Queensland, Brisbane, Australia; c Université de Lorraine, INRAE, LSE, Nancy, France; d Centre for Novel Agricultural Products, Department of Biology, University of York, York, UK

**Keywords:** Bioconcentration factor, hydroponics, hyperaccumulator

## Abstract

Toxic metals and metalloids, especially from anthropogenic sources, now pollute substantial areas of our planet. Phytoextraction is a proven technology with the potential to reduce metal/metalloid pollution, and where financially viable, recover valuable metals (‘phytomining’). Toward these aims, there has been a surge of publications over the last two decades. While important progress is being made, ongoing propagation of poor practice, and the resultant drain from funding sources, is hindering this promising research area. This includes mis-ascribing hyperaccumulator species, hydroponics with extremely high dose levels, misuse of Bioconcentration Factors, use of food or biomass crops with low accumulation for phytoextraction, the phenomenon of ‘template papers’ in which a known hyperaccumulator for element *X* is dosed with element *Y*, or a common weed species dosed with any variety of elements to make it ‘hyperaccumulate’. Here we highlight these misconceptions with the hope that this will help to: (i) disseminate accurate definitions for *in planta* metal accumulation; (ii) quash the propagation of poor practice by limiting the inflation of unnecessary publications *via* the practice of ‘template paper’ writing; (iii) be used by journal editors and reviewers to validate their reasoning to authors; and (iv) contribute to faster progress in delivering this technology to in-the-field practitioners.

## NOVELTY STATEMENT

In this note, we highlight some common misconceptions with the hope that this will help to disseminate accurate definitions for hyperaccumulation, promote the appropriate use of hydroponics, and limit template paper writing.

## Definition of hyperaccumulation

What is a metal/metalloid hyperaccumulator? A seemingly innocuous question, but one that is open to much debate because a universally accepted definition, based on verifiable molecular mechanisms, is lacking. Hyperaccumulators tend to concentrate metals or metalloids to >100 to 10,000 µg g^−1^ (or even higher) of the dry weight of the plant, and definitions for hyperaccumulation are phenomenological, justified by the orders-of-magnitude differences in shoot metal/metalloid concentrations observed between certain plant species and most others growing in the same soil environment (van der Ent *et al.* 2013; Baker and Brooks 1989; Baker and Whiting 2002). Numerous attempts have been made to operationally define hyperaccumulation, and recently a statistically-derived approach was used to validate historical threshold values for hyperaccumulation, largely confirming their veracity (Purwadi *et al.* 2023). Key characteriztics include a non-linear uptake response (*i.e.,* a non-linear accumulation as a function of the substrate concentration) to foliar metal/metalloid accumulation and hypertolerance to the metal or metalloid in question; but the fundamental mechanisms involved with hyperaccumulation differ greatly between elements and between species (van der Ent *et al.* 2015). The currently widely accepted definition of trace element hyperaccumulators are plants which, when growing in their natural habitat, rather than metal-amended artificial media, contain the elemental concentrations in excess of 100 µg g^−1^ cadmium (Cd), thallium (Tl) or selenium (Se); 300 µg g^−1^ cobalt (Co) or copper (Cu); 1000 µg g^−1^ nickel (Ni), arsenic (As), or Rare Earth Elements (REEs); 3000 µg g^−1^ zinc (Zn); and 10,000 µg g^−1^ manganese (Mn) dry weight shoot tissue (Reeves 2003; van der Ent *et al.* 2013; Purwadi *et al.* 2023). Govaerts *et al.* (2021) reported 342,953 vascular plant species but Reeves *et al.* (2018) listed only 721 as hyperaccumulators, which means that currently, hyperaccumulator species make up only ∼0.21% of known vascular species.

Despite flaws in defining hyperaccumulators, current hyperaccumulator species, and existing definitions, are well-described; mis-ascribing hyperaccumulator species should not readily occur; however, this is not the case. Here, we describe the main ways by which studies have inadvertently, or incorrectly, defined non-hyperaccumulators as hyperaccumulators. Note: we have deliberately not cited examples where terms have been misused as the purpose of this Note is to inform, not expose misguided mistakes. The incorrect usages of the term ‘hyperaccumulator’ includes where ‘hyperaccumulator’ is used to describe a species that supposedly hyperaccumulates several different metals indiscriminately, when in fact true hyperaccumulator species seldom hyperaccumulate more than one or two metals (van der Ent *et al.* 2013). Studies often then proceeded to describe the use of these species to remediate metals or metalloids that they do not hyperaccumulate. Moreover, some studies summarize data using maximum, or even outlier values, rather than mean metal or metalloid shoot concentration; or summarize results as hyperaccumulation when actual values were below the threshold metal concentrations; or use hydroponics and/or chelators to artificially assist uptake, or report only bioconcentration and translocation factors (see below).

## Artificial pseudo-hyperaccumulation from hydroponic growth systems

Hydroponic cultivation is a powerful and extremely useful experimental system for investigating metal/metalloid hyperaccumulator plants (van der Ent *et al.* 2024). However, it must be used with caution as almost any plant can be made to ‘hyperaccumulate’ if dose-levels are sufficiently high (for example dose rates of Cd in excess of 100 µM). This artificially high concentration leads to the disappearance of the characteristic differences between hyperaccumulators and non-accumulators due to saturation of the root-to-shoot translocation in the hyperaccumulator, or of the sequestration capacity in the non-accumulator root. Moreover, the experimental exposure times used are often short (hours to just a few days), giving a false impression that the plant will tolerate these concentrations over its lifecycle, when in fact in the longer term it will die. There might be good reasons for exposing (hyperaccumulator) plants to very high concentrations of a trace element to demonstrate hypertolerance, but this methodology cannot be used to claim hyperaccumulation. In the case of Cd, exposure levels of more than 5–10 µM in solution to achieve foliar concentrations of >100 µg g^−1^ are highly suspect. Similarly for Zn, 10–30 µM in solution to achieve foliar concentrations of >3000 µg g^−1^ Zn. Moreover, all known Cd hyperaccumulators grow in nature on soils with at least 100 times more Zn than Cd. Therefore, testing a species by supplying it with 10 µM Cd in the absence of 1000 µM Zn not only gives a false impression that it is a genuine Cd hyperaccumulator capable of high Cd uptake in the presence of Zn, but also has no practical use in phytoextraction of typical Zn-Cd contaminated soils. There can be real value in exposing numerous plant species to toxic levels of metals/metalloid, for comparative purposes, for tolerance screening, or indeed to discover genuine hyperaccumulating species; but exposure levels should be kept relatively low (<30 µM for most transition elements such as Ni or Zn) for testing tissue accumulation or higher (>100 µM) if testing tolerance (van der Ent *et al.* 2024). The ultimate test of whether a plant species is a genuine hyperaccumulator involves growing a test species on a natural soil enriched in the element of interest, as species such as *Arabidopsis halleri* can achieve >50,000 μg g^−1^ foliar Zn when growing on natural soil with just 340 μg g^−1^ Zn (Stein *et al.* 2017).

## Use of BCFs to describe hyperaccumulators

Hydroponic-based experiments in particular often report ‘high’ (>1) Bioconcentration Factors (BCFs) and use them to conclude hyperaccumulator potential, when both root and shoot underlying concentrations are relatively low (<1000 μg g^−1^). This problem also applies to studies undertaken with artificially-contaminated soil (spiked with soluble metal salts such as Pb(NO_3_)_2_) in which extremely high (*e.g.,*>10,000 µg g^−1^ Pb) prevailing metal/metalloid concentrations result in some uptake by the plant. If used sensibly, there is nothing wrong with this approach *per se*, if concentrations are realistic, or if used in a comparative test to show the tolerance of a true metallophyte/hyperaccumulator with a closely-related non-tolerant species. It is important to note that for many elements, potential for plant accumulation declines quickly after spiking, therefore soils are best spiked, fertilized, and equilibrated at least 3 months, remixed, and then used in experiments. However, the reverse can also be true, for example a plant with an entirely physiologically normal 100 μg g^−1^ foliar Zn growing in a soil with 10 μg g^−1^ Zn would be a ‘hyperaccumulator’ if solely based on its BCF value.

## Use of food crops to remediate toxic metals

With advances in plant breeding, and synthetic biology techniques, compartmentalization could one day be used to effectively separate metals to specific plants tissues, and perhaps applied to segregate metal-accumulating biomass from the edible parts in food crop species. However, while fundamental studies are an integral component in the development of phytoextraction tools, the use of food crop species to accrue metals and metalloids for phytoextraction purposes needs careful consideration. Food crops such as Indian mustard (*Brassica juncea*) and sunflower (*Helianthus annus*) have often been used in phytoextraction studies with the justification that they are ‘hyperaccumulators’ and ‘fast growing’, and thus able to remediate more metal/metalloid. However, these plant species are not hypertolerant or even demonstrably tolerant, and indeed are frequently killed in the phytoextraction process. It is a moot point as to whether it matters if the plant is living or dead at the end of the growth period if the metal is in the biomass. However, many non-food biomass crops massively outpace these species in yield, and deliver similar, or higher metal tissue concentrations, without risk of contaminating human food chains. Compare *Brassica juncea* (3.6–5.8 t ha^−1^) with switchgrass (*Panicum virgatum*) attaining (14.0–27.0 t ha^−1^) for example (Mandal and Sinha 2004; Giannoulis *et al.* 2016), or better still use dicotyledonous tree species such as *Salix* spp., as all grasses are well-known ‘excluder-type’ plants with low levels of accumulation (Rabêlo *et al.* 2021). Plant breeding has been used to develop a rice (*Oryza sativa*) genotype with improved agronomic traits that accumulates ∼10–30 μg g^−1^ Cd dry shoot biomass and has darker colored grains to prevent contamination with edible white rice (Abe *et al.* 2017). The genes for high Cd (*qCdp7*; a proposed loss-of-function allele of *OsHMA3*), and the grain color gene (which is "related to the *Rc* gene") are linked (∼3 Mb apart), so recombination frequency is low, but this frequency needs to be measured in-the-field, and the wider risks of Cd-rich rice entering the food chain assessed. The majority of studies on toxic metal/metalloid accumulation in rice focus on developing lines that exclude toxic metal/metalloids from the grain (Jing *et al.* 2023).

Many aromatic plants for example mint (*Mentha* spp.) and lavender (*Lavandula* spp.) will grow on soils polluted with toxic levels of metals and metalloids. These soils cannot be used to grow food crops (where part of the plant is directly eaten), and the financially valuable aromatic oils can be separated from the metal-rich biomass and sold. However, compared to biomass crops, these species are relatively small and slower growing. Calculations are needed to determine if phytoextraction rates, to remediate to below legal limits, sites contaminated, to depth, with toxic metals/metalloids are within ‘reasonable’ time frames.

## Template papers: replacing species or metal(loid)

Given the number of vascular plant species and perhaps twelve (Cr, Mn, Co, Ni, Cu, Zn, As, Se, Cd, mercury (Hg), Tl, Pb) relevant metal and metalloid elements to test, it is theoretically possible to conduct millions of experiments in a hydroponic-based system alone! In many countries, the pressure to continually publish is intense (Rawat and Meena 2014), therefore, it is perhaps not surprising, given the simplicity, and likely publication success, that the literature is inundated with examples of what we call ‘template papers’. The authors use a paper where a known hyperaccumulator for element *X* is dosed with element *Y*, then repeat the methodology over and over, publishing for each different element tested using the same basic model. An exception could be when uptake of a chemically analogous element is used, for example Co in a known Ni hyperaccumulator (Homer *et al.* 1991), or Cd in a known Zn hyperaccumulator (Brown *et al.* 1995), or lithium (Li) uptake in a known sodium (Na) accumulating plant species (Nkrumah and van der Ent 2022). However, there is no reason to assume why a selenium (Se) hyperaccumulator could be a Hg hyperaccumulator, or a Mn hyperaccumulator a Cd hyperaccumulator. A variation on this approach is using a plant species not known to be a hyperaccumulator (typically a common weed species or a horticultural cultivar) and dosing it with any variety of elements. A commonality is the use of hydroponic or pot-based systems, artificially dosed with a metal/metalloid of choice. These conditions do not reflect the natural environment for that species, and therefore, there is no evolutionary or physiological basis to suspect that it might genuinely hyperaccumulate this element. The template paper will then report the levels of a number of stress-related enzyme activities, metabolites, chlorophyll, and biomass, all predictably, with a positive correlation with increased levels of metal-induced stress. An equation needlessly defining the BCF will be added to convey mathematical knowledge, and the paper will end with a phrase concluding that this species is ‘potentially’ (see below) suitable for phytoextraction of ‘low’, ‘medium’ or ‘high’, depending on the results, levels of the metal/metalloid tested. In many cases, the studies are, from a methodological point of view, sound. In fact, often the analytical work undertaken is highly comprehensive and cutting-edge. This then raises the question as to whether a scientific paper in this field should be judged solely on its scientific veracity (in terms of the methods used), or also on its originality and practical utility to plant science. Numerous examples could be cited, but we are keen to point out that the aim of this text is not to vilify past misunderstandings, but to promote better practice. An additional hope is that Notes such as this, provide guidance for reviewers to pass to journal editors, and downstream to authors of such template manuscripts.

**Some prevailing scientific dogma are:**

- The common, but unqualified, assertion that the species can be used to remediate metal/metalloid contaminated land without calculating removal rates per harvest of per year. To avoid presenting these calculations can be misleading. Often, when removal rates are estimated, based on predicted annual biomass production and metal uptake rates, they run into 1000s of years. This point is further explained in Figure 5 of Rabêlo *et al.* (2021) in which the phytoextraction yield of various grasses and genuine hyperaccumulators are compared.
- The use of ‘potential’ throughout a paper for a species when analysis within the paper concludes the species to be a non-hyperaccumulator; and the unqualified use of the term ‘cost-effective’. This is hyperbole that can be used to enhance the apparent impact of a paper (or indeed the worth of a research grant application) yet does nothing to enhance our scientific knowledge in the field.
- It is over a decade since the scientific community moved away from the use of metal chelating compounds (such as EDTA) for phytoextraction to liberate/complex metals from soil phases and promote plant uptake, such as Pb, following conclusive research demonstrating the chemical persistence (and moblity) of metal complexes and their toxicity in the environment (Meers *et al.* 2009), and yet the number of publications describing the use of chelating compounds for phytoextraction has not decreased.
- The absence of discussion on the predicted effects of introducing metal-rich plants on the local ecosystem *via* herbivory. In geologically metal-rich regions (for example ultramafic soils enriched in Ni), flora and fauna have evolved to withstand high lprevailing concentrations of metals, but what of anthropogenic contamination?
- Research papers with little or no discussion on the fate of the metal-rich biomass that will be produced from the proposed phytoextraction. Whereas it might be financially viable to recover relatively high-value metals such as Ni or Co in phytomining, what to do with the waste produced of the lower value, and often more toxic, metals and metalloids such as Cd, Zn and As?

## Forward-focused research

Above, we have detailed misunderstandings and poor practice in the phytoextraction research area. However, collectively, research in this field has built an enormously valuable body of scientific evidence. We must now move from pot to field-based studies to establish parameters for industry stakeholders to assess project feasibility; develop financially viable uses for metal-rich biomass, particularly for low-value metals and metalloids; and metal recovery and concentration techniques. More molecular biology and biochemistry-based research is needed to understand the biology behind metal uptake and *in planta* accumulation. Following on from this is the, still lagging, adoption of synthetic biology techniques to engineer suites of high-biomass plants that specifically accumulate metals of our choosing. Metal pollution is affecting people’s lives and the health of our planet. We must not squander our resources on replicating what we already know, but look forward, and address the challenges preventing the use of this promising technology in the future. There are surely numerous, genuine hyperaccumulator plant species for a whole range of different elements still to be found, and if the rate of discovery through X-ray fluorescence (XRF) scanning of herbarium specimens (van der Ent *et al.* 2019) is anything to go by, the most exciting discoveries are still to be made.

## References

[CIT0001] Abe T, Ito M, Takahashi R, Honma T, Sekiya N, Shirao K, Kuramata M, Murakami M, Ishikawa S. 2017. Breeding of a practical rice line ‘TJTT8’ for phytoextraction of cadmium contamination in paddy fields. Soil Sci Plant Nutr. 63(4):388–395. doi: 10.1080/00380768.2017.1345598.

[CIT0002] Baker AJM, Brooks RR. 1989. Terrestrial higher plants which hyperaccumulate metallic elements—a review of their distribution, ecology and phytochemistry. Biorecovery. 1:81–126.

[CIT0003] Baker AJM, Whiting SN. 2002. In search of the Holy Grail – a further step in understanding metal hyperaccumulation? New Phytol. 155(1):1–4. doi: 10.1046/j.1469-8137.2002.00449_1.x. 33873303

[CIT0004] Brown SL, Chaney RL, Angle JS, Baker AJM. 1995. Zinc and Cadmium uptake by hyperaccumulator *Thlaspi caerulescens* grown in nutrient solution. Soil Sci Soc Am J. 59(1):125–133. doi: 10.2136/sssaj1995.03615995005900010020x. 22276881

[CIT0005] Giannoulis KD, Karyotis T, Sakellariou-Makrantonaki M, Bastiaans L, Struik PC, Danalatos NG. 2016. Switchgrass biomass partitioning and growth characteristics under different management practices. NJAS - Wageningen J Life Sci. 78(1):61–67. doi: 10.1016/j.njas.2016.03.011.

[CIT0007] Govaerts R, Nic Lughadha E, Black N, Turner R, Paton A. 2021. The world checklist of vascular plants, a continuously updated resource for exploring global plant diversity. Sci Data. 8(1):215. doi: 10.1038/s41597-021-00997-6. 34389730 PMC8363670

[CIT0008] Homer FA, Morrison RS, Brooks RR, Clemens J, Reeves RD. 1991. Comparative studies of nickel, cobalt, and copper uptake by some nickel hyperaccumulators of the genus *Alyssum*. Plant Soil. 138(2):195–205. doi: 10.1007/BF00012246.

[CIT0009] Jing H, Yang W, Chen Y, Yang L, Zhou H, Yang Y, Zhao Z, Wu P, Zia-Ur-Rehman M. 2023. Exploring the mechanism of Cd uptake and translocation in rice: future perspectives of rice safety. Sci Total Environ. 897:165369. doi: 10.1016/j.scitotenv.2023.165369. 37433335

[CIT0012] Mandal KG, Sinha AC. 2004. Nutrient management effects on light interception, photosynthesis, growth, dry-matter production and yield of Indian mustard (*Brassica juncea*). J Agronomy Crop Science. 190(2):119–129. doi: 10.1046/j.1439-037X.2003.00083.x.

[CIT0013] Meers E, Qadir M, de Caritat P, Tack FMG, Du Laing G, Zia MH, Saifullah. 2009. EDTA-assisted Pb phytoextraction. Chemosphere 74(10):1279–1291. doi: 10.1016/j.chemosphere.2008.11.007. 19121533

[CIT0014] Nkrumah PN, van der Ent A. 2022. Possible accumulation of critical metals in plants that hyperaccumulate their chemical analogues? Sci Total Environ. 878:162791. doi: 10.1016/j.scitotenv.2023.162791. 36907425

[CIT0016] Purwadi I, Erskine PD, Casey LW, van der Ent A. 2023. Recognition of trace element hyperaccumulation based on empirical datasets derived from XRF scanning of herbarium specimens. Plant Soil. 492(1–2):429–438. doi: 10.1007/s11104-023-06185-2.

[CIT0017] Rabêlo FHS, Vangronsveld J, Baker AJM, van der Ent A, Alleoni LRF. 2021. Are grasses really useful for the phytoremediation of potentially toxic trace elements? Front Plant Sci. 12:778275. doi: 10.3389/fpls.2021.778275. 34917111 PMC8670575

[CIT0018] Rawat S, Meena S. 2014. Publish or perish: where are we heading? J Res Med Sci. 19(2):87–89. 24778659 PMC3999612

[CIT0019] Reeves RD, Baker AJM, Jaffré T, Erskine PD, Echevarria G, van der Ent A. 2018. A global database for plants that hyperaccumulate metal and metalloid trace elements. New Phytol. 218(2):407–411. doi: 10.1111/nph.14907. 29139134

[CIT0020] Reeves RD. 2003. Tropical hyperaccumulators of metals and their potential for phytoextraction. Plant Soil. 249(1):57–65. doi: 10.1023/A:1022572517197.

[CIT0021] Stein RJ, Höreth S, de Melo JRF, Syllwasschy L, Lee G, Garbin ML, Clemens S, Krämer U. 2017. Relationships between soil and leaf mineral composition are element-specific, environment-dependent and geographically structured in the emerging model *Arabidopsis halleri*. New Phytol. 213(3):1274–1286. doi: 10.1111/nph.14219. 27735064 PMC5248639

[CIT0022] van der Ent A, Baker AJ, Reeves RD, Pollard AJ, Schat H. 2015. Commentary: toward a more physiologically and evolutionarily relevant definition of metal hyperaccumulation in plants. Front Plant Sci. 6:554. doi: 10.3389/fpls.2015.00554. 26257759 PMC4510833

[CIT0023] van der Ent A, Baker AJM, Reeves RD, Pollard AJ, Schat H. 2013. Hyperaccumulators of metal and metalloid trace elements: facts and fiction. Plant Soil. 362(1–2):319–334. doi: 10.1007/s11104-012-1287-3.

[CIT0024] van der Ent A, Echevarria G, Pollard AJ, Erskine PD. 2019. X-ray Fluorescence ionomics of herbarium collections. Sci Rep. 9(1):4746. doi: 10.1038/s41598-019-40050-6. 30894553 PMC6426943

[CIT0025] van der Ent A, Kopittke PM, Schat H, Chaney RL. 2024. Hydroponics in physiological studies of trace element tolerance and accumulation in plants focussing on metallophytes and hyperaccumulator plants. Plant and Soil. In Press. 10.1007/s11104-024-06537-6

